# LIF-VSR: A Lightweight Framework for Video Super-Resolution with Implicit Alignment and Attentional Fusion

**DOI:** 10.3390/s26020637

**Published:** 2026-01-17

**Authors:** Songyi Zhang, Hailin Zhang, Xiaolin Wang, Kailei Song, Zhizhuo Han, Zhitao Zhang, Wenchi Cheng

**Affiliations:** 1 School of Telecommunications Engineering, Xidian University, Xi’an 710071, China; zhangsongyi@stu.xidian.edu.cn (S.Z.); hlzhang@xidian.edu.cn (H.Z.); 2 Hebei Far East Communication Engineering Co., Ltd., Shijiazhuang 050200, China; wangxiaolin@hbfec.com.cn (X.W.); songkailei@hbfec.com.cn (K.S.); hanzhizhuo@hbfec.com.cn (Z.H.); zhangzhitao@hbfec.com.cn (Z.Z.); 3 Hebei Private Network Communication Technology Innovation Center, Shijiazhuang 050200, China

**Keywords:** video super-resolution, lightweight model, implicit deformable alignment, sparse attention fusion

## Abstract

Video super-resolution (VSR) has advanced rapidly in enhancing video quality and restoring compressed content, yet leading methods often remain too costly for real-world use. We present LIF-VSR, a lightweight, near-real-time framework built with an efficiency-first philosophy, comprising economical temporal propagation, a new neighboring-frame fusion strategy, and three streamlined core modules. For temporal propagation, a uni-directional recurrent architecture transfers context through a compact inter-frame memory unit, avoiding the heavy compute and memory of multi-frame parallel inputs. For fusion and alignment, we discard 3D convolutions and optical flow, instead using (i) a deformable convolution module for implicit feature-space alignment, and (ii) a sparse attention fusion module that aggregates adjacent-frame information via learned sparse key sampling points, sidestepping dense global computation. For feature enhancement, a cross-attention mechanism selectively calibrates temporal features at far lower cost than global self-attention. Across public benchmarks, LIF-VSR achieves competitive results with only 3.06 M parameters and a very low computational footprint, reaching 27.65 dB on Vid4 and 31.61 dB on SPMCs.

## 1. Introduction

The demand for high-quality, high-resolution video content is steadily increasing in applications such as surveillance, medical imaging, and mobile multimedia. Video super-resolution (VSR), which reconstructs a high-resolution sequence from a low-resolution one, plays a crucial role in these domains. Unlike Single-Image Super-Resolution (SISR) [1,2,3], which processes frames independently, VSR must simultaneously address cross-frame motion, alignment, and temporal fusion. Naively applying SISR frame-by-frame ignores temporal cues, resulting in temporal inconsistency and artifact accumulation. Consequently, the effective integration of alignment and fusion remains a central challenge in VSR [4,5,6].

To utilize temporal information effectively, existing VSR methods typically rely on either explicit motion estimation (e.g., optical flow) or implicit alignment (e.g., deformable convolution). However, flow-based approaches often incur substantial computational costs and are prone to artifacts when estimation fails in occluded regions. On the other hand, while recurrent frameworks have shown promise for efficient temporal propagation, current state-of-the-art designs often suffer from heavy memory pressure due to multi-frame parallel processing or quadratic complexity arising from global attention mechanisms. These computational bottlenecks hinder the practical deployment of VSR models on resource-constrained devices.

To address these challenges, we present LIF-VSR, a lightweight framework tailored for near real-time deployment. Our design centers on *efficient temporal propagation*, *sparse information aggregation*, and *low-cost feature enhancement*. Specifically, we propose a unidirectional recurrent architecture that maintains a minimal state, avoiding the overhead of multi-frame parallel inputs. For alignment and fusion, we introduce Implicit Deformable Alignment (IDA) and Sparse Attention Fusion (SAF). IDA performs cross-frame alignment without optical-flow priors by adaptively regressing sparse sampling offsets. SAF efficiently aggregates temporal cues using a small set of learnable key sampling points, transforming the quadratic complexity of global attention into a near-linear cost. Furthermore, a Temporal Cross Calibration (TCC) mechanism targets and calibrates features via cross-attention, where shallow features of the center frame serve as queries to enhance fused deep features.

Extensive experiments show that LIF-VSR achieves state-of-the-art performance on benchmarks such as Vid4 and SPMCs. Crucially, with only 3.06 M parameters and 17.60 GFLOPs, our model reduces the parameter count by approximately 50% compared to BasicVSR, offering a superior accuracy–efficiency trade-off.

We summarize our key contributions in three main points:

- We propose a minimal-state, unidirectional recurrent propagation scheme coupled with sparse neighboring-frame fusion, which effectively models temporal context without multi-frame parallelism, significantly reducing memory and compute.
- We introduce implicit deformable alignment and sparse attention fusion that regress a small set of sampling offsets from feature correlations to achieve cross-frame alignment and information aggregation without optical-flow priors.
- We adopt an RCAN-based lightweight backbone and propose a cross-attention-driven Temporal Cross Calibration mechanism that uses the center frame as a query to directionally align and enhance temporal features, substantially improving high-frequency detail.

## 2. Related Work

The goal of video super-resolution is to restore a high-resolution sequence from a low-resolution video by leveraging temporal information across frames—a key advantage over single-image super-resolution (SISR). This approach enables the recovery of more intricate details and ensures temporal smoothness. This section begins with a concise overview of the advancements in SISR, followed by a detailed examination of the core components of VSR, including its alignment strategies, network frameworks, and fusion/enhancement mechanisms.

### 2.1. Single-Image Super-Resolution

As a classic yet continually evolving research topic, Single-Image Super-Resolution is central to the field of computer vision. Beyond early interpolation and reconstruction methods, deep learning ushered in rapid progress: from the pioneering SRCNN [1] and deeper networks such as VDSR [7] to residual/attention-based designs like EDSR [2] and RCAN [3], SISR performance (e.g., PSNR) has been continuously advanced. More recently, Transformer-based architectures (e.g., SwinIR [8]) further improved long-range dependency modeling and feature representation. However, naively applying SISR frame-by-frame discards temporal cues, leading to flickering and artifact accumulation in the temporal dimension. Consequently, dedicated VSR models have been developed to explicitly leverage temporal information. Many state-of-the-art VSR systems still draw on successful SISR backbones, in particular, our model adopts an RCAN-style residual channel attention backbone for lightweight feature extraction.

### 2.2. Video Super-Resolution

The primary difficulty in VSR lies in effectively managing motion across frames to properly fuse their complementary information. Existing methods developed to address this issue can be broadly categorized as follows.

#### 2.2.1. Alignment-Based VSR

To utilize neighboring frames, one must first compensate for pixel displacements caused by object and camera motion.

**Explicit alignment.** A prevalent strategy is to first compute the optical flow between adjacent frames and then utilize this motion information to warp features towards the center frame for alignment. Representative methods include VESPCN [9], TOFlow [10], SOF-VSR [11] and EDVR [4], which often rely on pretrained flow estimators such as SPyNet [12] or modern variants (e.g., PWC-Net [13], RAFT [14]). While explicit motion compensation is intuitive and can handle large displacements, its effectiveness hinges on flow accuracy; flow errors lead to warping artifacts and blur. Moreover, accurate flow estimation is computationally heavy and may hinder real-time deployment.

**Implicit alignment.** To circumvent these issues, implicit, end-to-end alignment in feature space has gained traction. Deformable convolutions (DCN) [15,16] are a mainstream solution: TDAN [17] and BasicVSR++ [6] show that learning sparse sampling offsets enables flexible, content-adaptive aggregation for alignment, offering lower cost and better robustness to complex, non-rigid motion. Deformable 3D convolution extends offset learning to the spatiotemporal domain, enabling content-adaptive sampling across space–time [18], while lightweight kernelizations cut FLOPs and parameters—together boosting efficiency without degrading alignment fidelity [19]. Our Implicit Deformable Alignment (IDA) follows this efficient paradigm, targeting precise alignment under a lightweight budget.

#### 2.2.2. Recurrent VSR Frameworks

Based on their temporal processing methodology, VSR frameworks are typically divided into two main paradigms: sliding-window and recurrent. The sliding-window paradigm operates on fixed-size groups of frames (e.g., 5 or 7) simultaneously. While this approach is adept at capturing local temporal relationships, it suffers from poor scalability in terms of memory and computational cost when applied to longer video sequences. Recurrent frameworks address long-range temporal modeling more efficiently by processing frames sequentially and propagating a hidden state. FRVSR [20], BasicVSR [5], and BasicVSR++ [6] are representative: they aggregate past information via recurrent feature propagation, enabling constant memory/compute regardless of video length—well-suited to efficient and near real-time applications. In addition, RBPN [21] explores multi-stage back-projection with recurrence. Lightweight designs (FDDCC-VSR [19], LightVSR [22], RASVSR [23]) illustrate that careful architectural bottlenecks can retain accuracy without prohibitive compute. LIF-VSR builds upon this efficient unidirectional recurrent scheme, with our contributions focusing on lightweight and innovative alignment, fusion, and enhancement within the recurrent cell.

#### 2.2.3. Fusion and Enhancement in VSR

Aligned multi-frame features must be fused effectively to yield high-quality reconstructions. Early methods either concatenate features before applying convolutions or use 3D convolutions for joint spatiotemporal modeling; the former is often insufficiently adaptive, whereas the latter is computationally expensive. To more selectively exploit temporal cues, attention mechanisms have been introduced into VSR. While Transformer-based designs leverage global self-attention to effectively model long-range spatiotemporal dependencies, their quadratic complexity with respect to the number of tokens makes this approach computationally prohibitive for high-resolution inputs [24]. Despite their strong global context modeling [24,25], the cost remains substantial. Complementary frequency-domain processing has recently been shown to recover periodic textures and subtle high-frequency details that local convolutions may miss [26]. Motivated by these observations, LIF-VSR emphasizes efficient fusion and enhancement via sparse, cross-attentional designs that mitigate computational bottlenecks while preserving—and often improving—reconstruction quality.

## 3. Proposed Method

**Figure 1:**
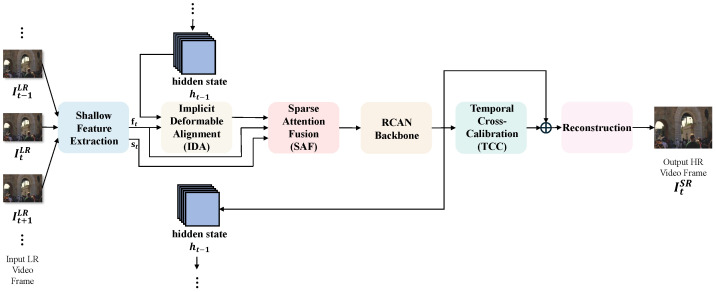
Overall architecture of the LIF-VSR framework.

We pursue a lightweight design guided by three principles: *minimal state*, *sample-on-demand*, and *directional calibration*. Concretely, LIF-VSR decomposes video super-resolution into (i) a unidirectional recurrent framework for sequence processing, (ii) temporal propagation via implicit deformable alignment and sparse fusion without optical flow, and (iii) feature enhancement and image reconstruction based on a compact RCAN backbone and cross-attentional calibration. This factorization preserves temporal coherence and high-frequency fidelity under tight parameter and compute budgets. Figure 1 illustrates the overall architecture of LIF-VSR.

### 3.1. Overall Architecture

Given a low-resolution (LR) sequence ${\left\{I_{t}^{\mathrm{LR}}\right\}}_{t=1}^{T}$, LIF-VSR processes frames sequentially and maintains a compact hidden state $h_{t-1}\in R^{C\times H\times W}$ to carry historical context. In contrast to sliding-window parallel schemes, the memory footprint is governed mainly by the spatial and channel dimensions rather than the temporal window length.

At each time step, we first extract shallow features for the current frame $f_{t}=\phi_{\mathrm{cur}}\left(I_{t}^{\mathrm{LR}}\right)$ and build a neighborhood summary $s_{t}=\phi_{\sup}\left(\mathrm{neigh}\left(I_{t}^{\mathrm{LR}}\right)\right)$ using mirrored boundary indexing. We then align and fuse historical and neighboring information:

(1) $$h_{t-1}^{\prime }=\mathrm{IDA}\left(h_{t-1},\;f_{t}\right),\;f_{t}^{\mathrm{fus}}=\mathrm{SAF}\left(f_{t},\;s_{t},\;h_{t-1}^{\prime }\right).$$

The fused feature is distilled by a lightweight RCAN backbone and calibrated via cross-attention using the current-frame shallow feature as the query:

(2) $$d_{t}=\mathrm{RCAN}\;\left(f_{t}^{\mathrm{fus}}\right),\;z_{t}=\mathrm{TCC}\;\left(f_{t},\;d_{t}\right),\;h_{t}=\mathrm{detach}\left(d_{t}\right).$$

The reconstruction head upsamples by two successive pixel-shuffle stages and adds an LR bypass:

(3) $$I_{t}^{\mathrm{SR}}=\mathrm{Upsample}\left(z_{t}\right)\;+\;{\mathrm{Up}}_{\mathrm{bilinear}}\;\left(I_{t}^{\mathrm{LR}}\right).$$

From a complexity viewpoint, the recurrent paradigm confines the dominant memory and latency terms to single-frame tensors (C,H,W) and avoids the bandwidth pressure of multi-frame stacks and 3D convolutions.

To formalize this workflow, we summarize the entire recurrent inference process in Algorithm 1. The subsequent sections will elaborate on the core components: IDA, SAF, and TCC.
**Algorithm 1** LIF-VSR Recurrent Inference Algorithm**Require:** Low-resolution video sequence $LQs={\left\{I_{t}^{\mathrm{LR}}\right\}}_{t=1}^{T}$**Ensure:** High-resolution video sequence $SRs={\left\{I_{t}^{\mathrm{SR}}\right\}}_{t=1}^{T}$1:Initialize hidden state $h_{0}\leftarrow 0$2:Initialize an empty output list SRs←[]3:**for** t←1toT **do**4:    $f_{t}\leftarrow \phi_{\mathrm{cur}}\left(I_{t}^{\mathrm{LR}}\right)$         ▹ Extract shallow features for the current frame5:    **if** t>1 **then**6:        $h_{t-1}^{\prime }\leftarrow \mathrm{IDA}\left(h_{t-1},f_{t}\right)$    ▹ Align the hidden state from the previous step7:    **else**8:        $h_{t-1}^{\prime }\leftarrow h_{0}$            ▹ No history for the first frame9:    **end if**10:    $s_{t}\leftarrow \phi_{\sup}\left(\mathrm{neigh}\left(I_{t}^{\mathrm{LR}}\right)\right)$      ▹ Extract features from neighboring frames11:    $f_{t}^{\mathrm{fus}}\leftarrow \mathrm{SAF}\left(f_{t},s_{t},h_{t-1}^{\prime }\right)$      ▹ Fuse current, neighbor, and historical info12:    $d_{t}\leftarrow \mathrm{RCAN}\left(f_{t}^{\mathrm{fus}}\right)$         ▹ Extract deep features via the backbone13:    $z_{t}\leftarrow \mathrm{TCC}\left(d_{t},\mathrm{query}=f_{t}\right)$       ▹ Calibrate with cross-attention14:    $I_{t}^{\mathrm{SR}}\leftarrow \mathrm{Upsample}\left(z_{t}\right)+{\mathrm{Up}}_{\mathrm{bilinear}}\left(I_{t}^{\mathrm{LR}}\right)$   ▹ Reconstruct the HR image15:    Append $I_{t}^{\mathrm{SR}}$ to the SRs list16:    $h_{t}\leftarrow \mathrm{detach}\left(d_{t}\right)$      ▹ Update the hidden state for the next frame17:**end for**18:**return** SRs

### 3.2. Temporal Propagation, Alignment and Fusion

This component aligns the historical state to the current content and fuses temporal cues efficiently, all in the feature domain without optical flow or warping.

#### 3.2.1. Implicit Deformable Alignment (IDA)

**Figure 2:**
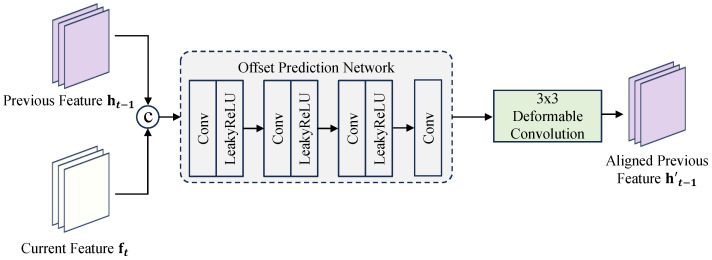
The internal structure of the Implicit Deformable Alignment (IDA) module. Unlike flow-guided approaches, IDA directly regresses offsets from feature correlations, ensuring lightweight and stable alignment.

To align the historical state $h_{t-1}$ with the current frame $f_{t}$, we propose the Implicit Deformable Alignment (IDA) module (see Figure 2). While previous methods such as EDVR [4] and BasicVSR++ [6] also employ deformable convolutions, they typically rely on *cascaded pyramidal processing* or *explicit optical flow guidance* (e.g., using SPyNet [12]) to predict offsets. These approaches incur significant computational overhead and are prone to instability when optical flow estimation fails in occluded or textureless regions.

In contrast, our IDA is designed for efficiency and stability. It discards the heavy pre-trained flow network and instead learns to regress offsets directly from local feature correlations. Specifically, conditioned on the concatenation $\left[h_{t-1},f_{t}\right]$, a lightweight convolutional head predicts the residual sampling offsets $\Delta p_{k}$ and modulation masks $m_{k}$. The alignment is performed via:

(4) $$y\left(p_{0}\right)\;=\;\sum_{k=1}^{K}w_{k}\;\left[\;x\;\;\left(p_{0}+p_{k}+\Delta p_{k}\right)\;\right]\;m_{k},$$

where $x=h_{t-1}$.

This design offers three key advantages over prior DCN-based methods:

1. **Direct Feature Regression:** By bypassing explicit flow warping, IDA avoids the error propagation associated with flow estimation artifacts.
2. **Computational Efficiency:** IDA eliminates the need for bulky flow estimators. Specifically, it reduces the parameter count of the alignment module by approximately 70% compared to the standard pre-trained SPyNet [12] used in flow-guided counterparts.
3. **Stability Mechanism:** The offsets are learned end-to-end within the reconstruction objective, allowing the network to suppress sampling in unreliable regions via the modulation masks $m_{k}$, thereby providing superior robustness against large motions and occlusions.

#### 3.2.2. Sparse Attention Fusion (SAF)

**Figure 3:**
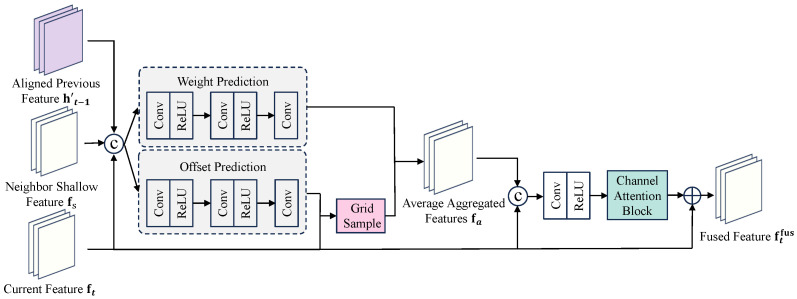
Illustration of the Sparse Attention Fusion (SAF) module. It learns sparse offsets and weights to aggregate multi-source temporal information.

Dense 3D convolutions incur high memory bandwidth, and global self-attention scales quadratically with spatial positions, which is prohibitive at mid/high resolutions. We reformulate global correspondence as sparse keypoint sampling and weighted aggregation (see Figure 3).

For the current feature and a candidate temporal branch (neighboring or aligned history), an offset head predicts *K* offsets ${\left\{\Delta p_{k}\right\}}_{k=1}^{K}$ and a weight head yields ${\left\{\alpha_{k}\right\}}_{k=1}^{K}$ with Softmax normalization. First, for each temporal branch (e.g., neighbor features or the aligned hidden state), we compute an aggregated feature, denoted as $\bar{a}$, using a sparse sampling mechanism. At a location $p_{0}$, this is calculated as:

(5) $$\bar{a}\left(p_{0}\right)\;=\;\sum_{k=1}^{K}\alpha_{k}\;\left[\;g\;\;\left(p_{0}+\Delta p_{k}\right)\;\right],$$

where g is the feature map of the temporal branch being sampled. After computing the aggregated feature ${\bar{a}}_{i}$ for each of the *B* temporal branches, we concatenate them with the central feature $f_{t}$. This combined tensor is subsequently fed into a fusion block Γ(·) (a 1×1 convolution followed by channel attention) to produce the final fused feature for the current timestep, $f_{t}^{\mathrm{fus}}$:

(6) $$f_{t}^{\mathrm{fus}}\;=\;\Gamma \;\left(\mathrm{Concat}\left[f_{t},\;{\bar{a}}_{1},\;\ldots ,\;{\bar{a}}_{B}\right]\right).$$

### 3.3. Feature Enhancement and Reconstruction

#### 3.3.1. Lightweight RCAN Backbone

**Figure 4:**
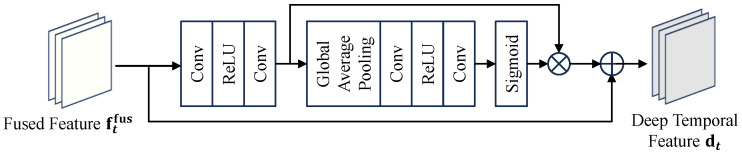
The architecture of the Residual Channel Attention Block (RCAB).

We adopt a compact RCAN as our feature extraction backbone. It serves as a standardized baseline, ensuring that performance gains are primarily derived from our proposed temporal alignment and fusion schemes rather than a heavier spatial backbone. It is composed of several residual groups; each group stacks multiple RCABs (see Figure 4). For an input u, the channel rescaling in an RCAB reads

(7) $$s\;=\;\sigma \;\left(W_{2}\;\delta \left(W_{1}\;\mathrm{GAP}\left(\phi \left(u\right)\right)\right)\right),\;\mathrm{RCAB}\left(u\right)\;=\;u\;+\;s⊙\phi \left(u\right),$$

where GAP denotes global average pooling, δ(·) is ReLU, σ(·) is Sigmoid, ϕ(·) denotes a convolutional transform, and ⊙ is channel-wise multiplication.

Group-level residual connections stabilize deep optimization. With moderate channels (e.g., C=64), the $O(C^{2}HW)$ cost remains tractable while effectively boosting texture/structure responses.

#### 3.3.2. Temporal Cross Calibration (TCC)

**Figure 5:**
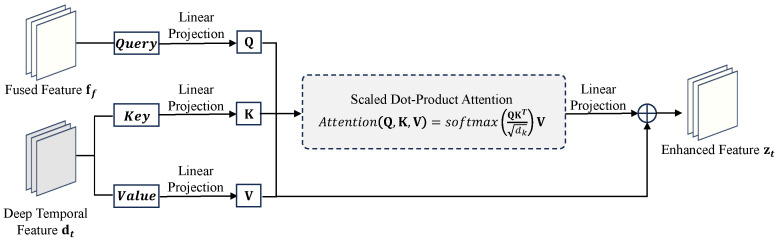
The cross-attention mechanism in the Temporal Cross Calibration (TCC) module. Shallow features serve as the Query to calibrate the deep features.

Deep temporal fusion may dilute high-frequency cues of the current frame, whereas self-attention on $d_{t}$ alone is costly. We instead perform cross-attention with the shallow current feature as the query and the deep temporal feature as key/value (see Figure 5).

The TCC module is built upon a scaled dot-product attention mechanism, whose output acts as a calibration signal that is subsequently added back to the original deep feature $d_{t}$ via a residual connection to produce the final calibrated feature, denoted as $z_{t}$. This process is formulated as:

(8) $$z_{t}\;=\;d_{t}\;+\;\Psi \;\left(\mathrm{Attention}(Q,K,V)\right),$$

where $\mathrm{Attention}\left(Q,K,V\right)=\mathrm{softmax}\;\left(\frac{QK^{⊤}}{\sqrt{d_{k}}}\right)V$. The Query, Key, and Value are derived as $Q=W_{q}f_{t}$, $K=W_{k}d_{t}$, and $V=W_{v}d_{t}$, with Ψ(·) representing a final linear projection. This design emphasizes content most relevant to the current frame while avoiding the quadratic complexity and memory of global self-attention on $d_{t}$.

It is worth noting that TCC is explicitly designed as a residual module: $z_{t}=d_{t}+\Psi \left(\ldots \right)$. Since the main feature stream $d_{t}$ is derived from the recurrent hidden state which aggregates long-term temporal context, it inherently maintains strong temporal stability. The attention map acts solely as a high-frequency calibration signal. Consequently, this residual structure ensures that the single-frame guidance enhances spatial details without disrupting the underlying temporal coherence of the video sequence.

#### 3.3.3. Upsampling and Image Reconstruction

The reconstruction head employs two pixel-shuffle stages, each scaling by ×2. Let ${\tilde{z}}_{t}^{\left(1\right)}$ and ${\tilde{z}}_{t}^{\left(2\right)}$ be the channel-expanded tensors prior to shuffling, and let $\Pi_{\times 2}$ denote channel-to-space rearrangement:

(9) $$z_{t}^{\left(1\right)}\;=\;\Pi_{\times 2}\;\left({\tilde{z}}_{t}^{\left(1\right)}\right),\;z_{t}^{\left(2\right)}\;=\;\Pi_{\times 2}\;\left({\tilde{z}}_{t}^{\left(2\right)}\right).$$

A high-resolution convolutional head maps $z_{t}^{\left(2\right)}$ to RGB, and an LR bilinear bypass is added as residual:

(10) $$I_{t}^{\mathrm{SR}}\;=\;\Phi \;\left(z_{t}^{\left(2\right)}\right)\;+\;{\mathrm{Up}}_{\mathrm{bilinear}}\;\left(I_{t}^{\mathrm{LR}}\right).$$

where Φ(·) denotes the final HR convolution. Pixel-shuffle converts spatial enlargement into channel rearrangement, avoiding checkerboard artifacts and reducing the amount of computation performed at high resolution; the residual design enables the network to better concentrate on learning high-frequency details.

### 3.4. Dataset Description

We use REDS [27] for training and evaluate on Vid4 [9], SPMCs [28], and REDS4 [27]. All datasets are downsampled using bicubic interpolation (BI) to create LR-HR training pairs. REDS covers diverse scenes and strong/non-rigid motion, with a resolution around 720 p and typically 100 frames per sequence. We adopt the official training split (about 240 sequences), generate ×4 low-resolution (LR) inputs from high-resolution (HR) frames via bicubic downsampling. For data augmentation, we employ a set of geometric transformations, including vertical flips and random horizontal, as well as rotations by $90^{\circ }$.

For evaluation, Vid4 consists of four classic sequences, each with roughly 30 frames at standard-definition resolution. It contains moderate camera/object motion and many fine repetitive textures, and is commonly used to assess temporal consistency and detail fidelity. SPMCs includes 11 sequences (about 31 frames per sequence, resolution ≈960×540) spanning translation, scaling, panning/tilting, and noticeable parallax, thus emphasizing alignment robustness and temporal aggregation. REDS4 is a four-sequence subset selected from the REDS validation set, featuring fast motion, illumination changes, and motion blur—challenges that effectively gauge the generalization of lightweight methods in complex real-world scenarios.

### 3.5. Evaluation Metrics

The proposed system was implemented using the PyTorch 2.7 framework and trained on 8 NVIDIA GeForce RTX 4090 GPUs. For the training data, we extracted video clips of 30 frames in length. Input frames were randomly cropped to a resolution of 256×256 and augmented through random flips and rotations. Optimization uses Adam [29] with an initial learning rate of $1\times 10^{-4}$ and a Cosine Annealing with Warm Restarts schedule [30]. We used a batch size of 32 and trained the model for a total of $10^{5}$ iterations, which corresponds to approximately ten epochs over the dataset.

We report reconstruction quality using PSNR and SSIM. PSNR is computed from the mean squared error (MSE) between the reconstruction $\hat{I}$ and ground truth *I*:

(11) $$\mathrm{PSNR}=10\log_{10}\;\left(\frac{MAX_{I}^{2}}{\mathrm{MSE}}\right),\;\mathrm{MSE}=\frac{1}{N}\sum_{p=1}^{N}{\left({\hat{I}}_{p}-I_{p}\right)}^{2}.$$

Higher PSNR indicates lower distortion. SSIM measures perceptual similarity by comparing luminance, contrast, and structure, which is calculated as:

(12) $$\mathrm{SSIM}\left(x,y\right)=\frac{\left(2\mu_{x}\mu_{y}+C_{1}\right)\left(2\sigma_{xy}+C_{2}\right)}{\left(\mu_{x}^{2}+\mu_{y}^{2}+C_{1}\right)\left(\sigma_{x}^{2}+\sigma_{y}^{2}+C_{2}\right)},$$

where $\mu_{x}$ and $\mu_{y}$ represent the mean values of *x* and *y*, $\sigma_{x}^{2}$ and $\sigma_{y}^{2}$ denote their variances, $\sigma_{xy}$ is the covariance, and $C_{1},C_{2}$ are constants to stabilize the division. SSIM is bounded in [0,1], and higher values are better.

## 4. Results

### 4.1. Quantitative Comparison

We compare the performance of LIF-VSR with state-of-the-art (SOTA) VSR methods on Vid4, SPMCs, and REDS4 benchmarks. Table 1 summarizes the PSNR and SSIM scores.

As shown in Table 1:

- On **Vid4**, LIF-VSR achieves the highest PSNR of 27.65 dB, surpassing the second-best FDI-VSR by 0.36 dB. It also obtains a competitive SSIM of 0.8283.
- On **SPMCs**, our method secures the top spot in both metrics with a PSNR of 31.61 dB and an SSIM of 0.8874, outperforming L-VSR by a margin of 2.59 dB.
- On **REDS4**, LIF-VSR achieves a PSNR of 30.69 dB, ranking third behind FDI-VSR (31.11 dB) and L-VSR (30.71 dB).

### 4.2. Efficiency Analysis

Table 2 presents the model complexity in terms of parameter count and FLOPs (calculated on a 64×64 input with ×4 upscaling).

LIF-VSR contains 3.06 million parameters, which is approximately 49% of BasicVSR (6.29 M) and 44% of BasicVSR++ (7.03 M). In terms of computational cost, our model requires 17.60 GFLOPs per frame, which is lower than BasicVSR (26.26 GFLOPs) and EDVR-M (32.91 GFLOPs), and comparable to the lightweight DUF (16.90 GFLOPs).

### 4.3. Ablation Experiments

In this section, we conduct a comprehensive ablation study to verify the effectiveness of our proposed framework. We first analyze the contribution of each core component, followed by a detailed discussion on the sampling strategy within the Sparse Attention Fusion (SAF) module.

#### 4.3.1. Effectiveness of Core Components

We investigate the contribution of Implicit Deformable Alignment (IDA), Sparse Attention Fusion (SAF), and Temporal Cross Calibration (TCC) by incrementally adding them to the baseline. The quantitative results are reported in Table 3.

As observed in Table 3:

- **Baseline + IDA:** Introducing the IDA module increases the PSNR by 1.32 dB on Vid4 and 1.84 dB on SPMCs compared to the baseline, confirming that feature-space alignment significantly outperforms simple concatenation.
- **Baseline + IDA + SAF:** Adding the SAF module further improves PSNR by 0.39 dB on Vid4 and 0.66 dB on SPMCs, validating the efficacy of sparse temporal aggregation.
- **Full Model (+ TCC):** The inclusion of TCC yields the final performance of 27.65 dB on Vid4 and 31.61 dB on SPMCs, demonstrating the benefit of high-frequency calibration.

#### 4.3.2. Analysis of Sampling Granularity in SAF

The number of sampling points *K* in the SAF module determines the granularity of temporal aggregation. We interpret K=9 as the sparse equivalent of a standard 3×3 convolution kernel, which is a proven “sweet spot” in computer vision for capturing local geometric transformations. To verify this, we conducted an ablation study with K∈{4,9,25}, corresponding to 2×2, 3×3, and 5×5 receptive fields, respectively.

As shown in Table 4, reducing *K* to 4 results in a noticeable performance drop (0.18 dB on Vid4), indicating insufficient context aggregation. Conversely, increasing *K* to 25 yields only marginal gains (+0.03 dB) but increases computational complexity. Therefore, K=9 offers the optimal trade-off.

It is also worth noting that we employ *multi-group deformable attention* with G=16 groups. This means that while each head samples only 9 points, the model effectively aggregates information from 16×9=144 distinct spatial locations per position, providing a rich context summary without the prohibitive memory cost of dense global attention.

### 4.4. Visualization Analysis

**Figure 6:**
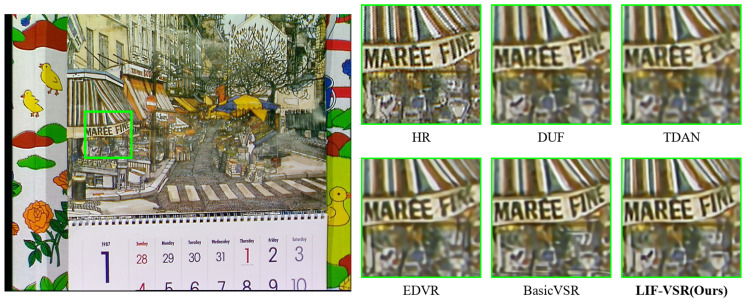
Qualitative comparison on the “Calendar” sequence from Vid4. Our LIF-VSR excels at restoring sharp text characters (e.g., “MAREE FINE”) and fine awning patterns.

**Figure 7:**
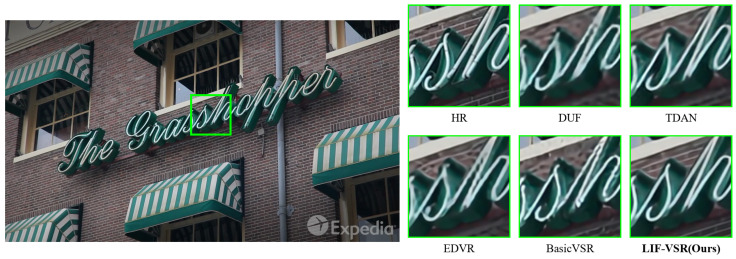
Qualitative comparison on a sequence from the SPMCs dataset. LIF-VSR demonstrates superior ability in preserving the geometry of the neon signage and the surrounding brick textures.

**Figure 8:**
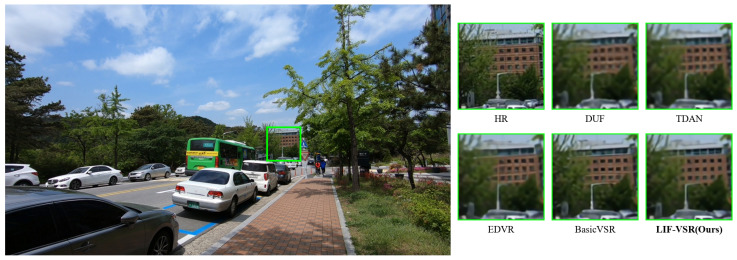
Qualitative comparison on a sequence from REDS4. LIF-VSR effectively handles the distant building details, resulting in significantly clearer window grids compared to other methods.

Visual comparisons on sequences from the Vid4, SPMCs, and REDS4 datasets are shown in Figure 6, Figure 7, and Figure 8, respectively.

In the “Calendar” sequence from Vid4 (Figure 6), methods such as DUF and TDAN produce blurred text where the characters are barely recognizable. In contrast, LIF-VSR effectively reconstructs the sharp edges of the text “MAREE FINE” and the distinct striped patterns on the awning, demonstrating superior recovery of high-frequency details.

For the sequence from SPMCs (Figure 7), which features a complex brick facade and neon signage, competing methods like EDVR and BasicVSR exhibit aliasing artifacts and blurring around the cursive letters. LIF-VSR maintains the structural integrity of the “Grasshopper” signage and separates the neon tubes from the background brick texture more clearly.

In the REDS4 example (Figure 8), the scene contains distant building structures that challenge temporal consistency. While other methods struggle to resolve the grid patterns of the windows, resulting in severe motion blur, LIF-VSR successfully restores the rectangular window shapes and sharp building edges, proving its robustness in handling complex outdoor scenes.

**Figure 9:**
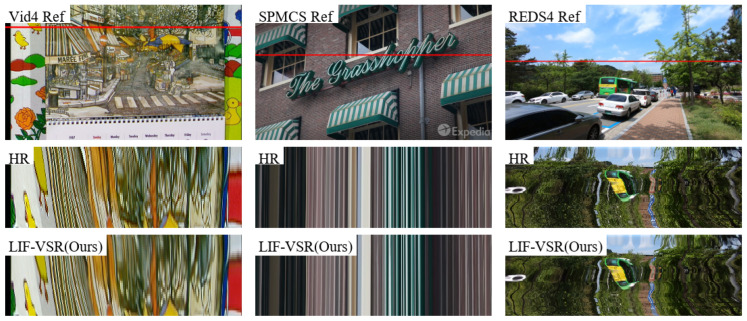
Temporal profile comparison across three datasets. The red lines in the reference frames indicate the sampling positions. The temporal profiles of LIF-VSR exhibit smooth, continuous structures that closely match the Ground Truth (HR), verifying that the proposed method maintains excellent temporal consistency.

To address the concern that the single-frame guidance in the TCC module might introduce temporal instability, we visualized the temporal profiles (vertical slices of the video volume over time) across the Vid4, SPMCS, and REDS4 datasets. As shown in Figure 9, the temporal trajectories generated by LIF-VSR are visually indistinguishable from those of the Ground Truth (HR).

Specifically, in the Vid4 and REDS4 samples, which contain complex motion (manifested as curved patterns in the temporal profile), LIF-VSR accurately reconstructs the continuous flow of pixels without exhibiting jagged artifacts or discontinuities. Similarly, in the static regions of the SPMCS sample, the straight vertical lines remain stable and sharp. This strong alignment with the HR reference confirms that the residual design of the TCC module enhances spatial details while strictly preserving the underlying temporal coherence of the video.

## 5. Discussion

The experimental results presented in Section 4 demonstrate that LIF-VSR achieves a superior balance between reconstruction quality and model complexity. As illustrated in Table 1, our method outperforms existing lightweight and prominent recurrent networks on the Vid4 and SPMCs benchmarks. A critical insight from our efficiency analysis is that high performance in VSR does not necessarily require heavy optical flow estimation or dense global attention. By reducing the parameter count to 3.06 M—approximately 50% of BasicVSR—LIF-VSR proves that a unidirectional recurrent scheme with minimalist state propagation is sufficient for capturing long-range temporal dependencies, provided that the alignment and fusion mechanisms are carefully designed.

The effectiveness of our design philosophy is further supported by the ablation study. The dominance of the Implicit Deformable Alignment (IDA) module in performance gains confirms that feature-space alignment is more robust than explicit motion compensation, particularly for complex, non-rigid motions. Furthermore, the success of the Sparse Attention Fusion (SAF) module validates our hypothesis that global attention is redundant for VSR. By selecting only a small set of key sampling points, SAF effectively aggregates relevant temporal cues without the quadratic complexity of full self-attention. The Temporal Cross Calibration (TCC) further refines these features by using the current frame’s high-frequency information to prevent the “smoothing out” effect.

Despite these promising results, LIF-VSR has limitations. The random memory access patterns inherent in deformable convolutions and sparse sampling may exhibit lower inference speedup on certain hardware accelerators compared to standard convolutions. Additionally, while our unidirectional scheme is efficient, extremely large or abrupt scene changes might challenge the propagation stability compared to bidirectional approaches.

## 6. Conclusions

In this paper, we addressed the efficiency bottleneck in video super-resolution by proposing LIF-VSR, a lightweight framework tailored for practical deployment. By synergizing Implicit Deformable Alignment, Sparse Attention Fusion, and Temporal Cross Calibration, we successfully eliminated the need for heavy optical flow estimation and dense attention mechanisms. Our method achieves state-of-the-art performance on standard benchmarks while maintaining a low computational footprint.

Future work will focus on two directions: exploring lightweight bidirectional propagation strategies to further handle complex scene transitions, and optimizing the inference latency of deformable operators on edge hardware devices.

## Figures and Tables

**Table 1:** Performance comparison of our LIF-VSR against SOTA methods on the task of 4× video super-resolution. The evaluation is conducted on the Vid4, SPMCs, and REDS4 benchmark datasets, with PSNR and SSIM as the primary metrics. The top three performing methods are highlighted: **first** (bold), second (underlined), and *third* (italic).

Method	Year	Vid4		SPMCs		REDS4	
		**PSNR**	**SSIM**	**PSNR**	**SSIM**	**PSNR**	**SSIM**
Bicubic	-	21.80	0.5246	25.67	0.7260	26.14	0.7292
VESPCN [9]	2017	25.09	0.7323	27.83	0.8230	29.29	0.8451
DUF [31]	2018	26.80	0.8134	30.10	0.8556	29.50	0.8283
DBPN [32]	2018	25.32	0.7362	27.92	0.8225	29.44	0.8475
RCAN [3]	2018	25.46	0.7404	28.36	0.8287	29.51	0.8489
PFNL [33]	2019	26.40	**0.8284**	28.66	0.8478	29.63	0.8502
SOF-VSR [11]	2020	26.02	0.7713	28.21	0.8324	29.73	0.8519
ZS-Mo [34]	2020	26.14	0.7974	28.80	0.8635	29.93	0.8634
TDAN [17]	2020	26.16	0.7821	28.51	0.8412	29.87	0.8533
D3Dnet [18]	2020	26.52	0.7993	28.78	0.8523	30.51	*0.8657*
FastDVDnet [35]	2021	26.14	0.7719	28.53	0.8465	29.57	0.8474
TMNet [36]	2021	26.23	0.8041	28.78	*0.8640*	29.91	0.8633
RSTT [25]	2022	26.20	0.7991	28.86	0.8634	30.11	0.8613
STDAN [37]	2023	26.28	0.8041	28.94	0.8687	29.98	0.8613
LRGAN [38]	2023	26.80	0.8149	28.95	0.8608	29.82	0.8383
L-VSR [22]	2025	*26.95*	0.8188	29.02	0.8583	30.71	**0.8780**
FDI-VSR [39]	2025	27.29	*0.8230*	*29.84*	0.8597	**31.11**	0.8674
**LIF-VSR (ours) **	2025	**27.65**	0.8283	**31.61**	**0.8874**	*30.69*	0.8566

**Table 2:** Comparison of model parameters and computational cost. All metrics are for a single LR frame input of size 64×64 and a scale factor of ×4.

Method	Input Size	Scale	Parameter (M)	Computational Cost Per Frame (GFLOPs)
BasicVSR++ [6]	64×64	×4	7.03	28.26
BasicVSR [5]	64×64	×4	6.29	26.26
DUF [31]	64×64	×4	5.82	16.90
EDVR-M [4]	64×64	×4	3.15	32.91
**LIF-VSR (ours)**	64×64	×4	3.06	17.60

**Table 3:** Ablation experiments of the core components of LIF-VSR on the Vid4, SPMCs, and REDS4 datasets. ‘✓’ indicates the module is active, while ‘×’ indicates it is deactivated.

Dataset	IDA	SAF	TCC	PSNR	SSIM
**Vid4**	×	×	×	25.81	0.7502
	✓	×	×	27.13	0.8115
	✓	✓	×	27.52	0.8248
	✓	✓	✓	**27.65**	**0.8283**
**SPMCs**	×	×	×	28.95	0.8133
	✓	×	×	30.79	0.8641
	✓	✓	×	31.45	0.8832
	✓	✓	✓	**31.61**	**0.8874**
**REDS4**	×	×	×	28.62	0.7989
	✓	×	×	30.08	0.8381
	✓	✓	×	30.56	0.8525
	✓	✓	✓	**30.69**	**0.8566**

**Table 4:** Ablation study on the number of sampling points (*K*) in the SAF module. Evaluated on Vid4 and SPMCs.

Sampling Points (*K*)	Params (M)	GFLOPs	Vid4 PSNR (dB)	SPMCs PSNR (dB)
K=4 (2×2)	3.04	17.42	27.47	31.42
K=9 (3×3) **(Default)**	3.06	17.60	27.65	31.61
K=25 (5×5)	3.12	18.15	**27.68**	**31.70**

## Data Availability

All datasets utilized in this research are publicly available. For training and evaluation, we used the Vid4 (https://people.csail.mit.edu/celiu/CVPR2011/videoSR.zip (accessed on 12 December 2025)), SPMCs (https://github.com/jiangsutx/SPMC_VideoSR (accessed on 12 December 2025)), and REDS4 (https://seungjunnah.github.io/Datasets/reds.html (accessed on 12 December 2025)) datasets, all of which are accessible at their respective URLs.
